# Does Size Matter? The Case of the Courtship Pyramids in Red Sea Ghost Crabs (*Ocypode saratan*)

**DOI:** 10.3390/ani11123541

**Published:** 2021-12-13

**Authors:** Reuven Yosef, Michal Korkos, Jakub Z. Kosicki

**Affiliations:** 1 Eilat Campus, Ben Gurion University of the Negev, Eilat 88000, Israel; 2 Rabin High School, 51 Yotam Street, Eilat 88104, Israel; michalkorkos1505@gmail.com; 3 Department of Avian Biology and Ecology, Adam Mickiewicz University, Poznań, Uniwersytetu Poznańskiego Str. 6, 61-614 Poznań, Poland; kubako@amu.edu.pl

**Keywords:** Red Sea, sexual selection, carapace size, sand pyramid

## Abstract

**Simple Summary:**

Male ghost crabs (*Ocypode* spp.) are known to build sand structures near the entrance to their burrows. It has always been assumed that the structure played a role in sexual selection and mate attraction. We hypothesized that the larger males would make relatively larger pyramids in order to enhance fitness. We studied Red Sea ghost crabs at Eilat, Israel, and found that the larger crabs actually did not put much effort into building pyramids, and it was the medium-sized crabs that invested the most in building the pyramids.

**Abstract:**

Display, wherein males attempt to maximize fitness by attracting sexually mature females to mate, is known to drive speciation by Sexual Selection. We researched the Red Sea Ghost Crab (*Ocypode saratan*; RSGC), in which males build display pyramids to attract females. The study was conducted at the beach in Eilat, Israel. At each session, we measured the height (in cm) of all pyramids and the dimensions (height, breadth; in cm) of the burrow entrance. We assumed that the size of the entrance represented the relative size of the carapace width of the occupant. The mean (± SE) entrance volume was 230.8 ± 11.7 cm, and the height of the pyramid was 11.8 ± 0.49 cm (*n* = 54). The results of our study did not support our hypothesis because we had expected to find a linear correlation between body size and pyramid height, i.e., the larger the male, the larger the pyramid. However, our results show that the largest males in the population either built small pyramids or not at all, and the cut-off of the larger crab’s body size appears to be around 350 cm^3^. We discovered a step-wise function in the data in that crabs with the smallest body size of ca. 250 cm^3^ constructed the highest pyramids, with a declining tendency between 250–350 cm^3^ and extremely low pyramids beyond 350 cm^3^. However, our findings need to be further studied with a stress on the ambiance and elucidate whether the habitats differ in temperature, humidity, prey-base, etc., before concluding as to why the larger males desist from building pyramids. This study underwrites the importance of studying the mating systems of the macro-fauna of the beaches that are fast disappearing owing to anthropogenic development.

## 1. Introduction

Since the theory of speciation and natural selection were co-proposed [1,2], the subject is still debated, and many subsequent models either support or refute their theory [3,4]. One of the major hypotheses in natural selection theory is that of speciation by Sexual Selection (SSS) [5], wherein it is argued that female preferences drive male traits, especially display, resulting in reproductive isolation [6].

Display, wherein males attempt to maximize fitness by attracting reproductive females to mate, is known to drive SSS [6]. The subject of display has been found to occur in a wide range of empirical studies such as quantity and quality of prey [7,8], color and ornamentation [9], vocalizations [10], ritual dance [11], chemical [12], and several other qualities that Zahavi collated under the “Handicap Principle” [13,14].

Trying to impress females to choose themselves in a lek mating system requires males to outdo their competition by being either more vocal or colorful, executing a more elaborate dance and/or having exemplary ornamentation [15]. Although this has not been clarified for any of the Ocypode species studied to date globally, from our observations in the field, we are convinced that the ghost crabs have a lek mating system, wherein the males display and build their pyramids in proximity to each other, and attract females from the surrounding areas of the beach [16]. Our speculations are supported indirectly by biased sex ratios in other ghost crabs and in New World fiddler crabs [17,18,19].

According to this, we researched a little-studied species of the genus *Ocypode*, Red Sea Ghost Crab (*O. saratan*; RSGC), in which males build display pyramids to attract females [20,21]. In spite of the two detailed studies of the RSGC, neither classified the mating system nor did they study whether body size of the adult male influenced pyramid size. Hence, we initiated a study on a sandy beach in Eilat, Israel, where the species is readily observed. We hypothesized that size would influence the final product and that the larger the male, the larger the height of the pyramid would be, in accordance with the Handicap Principle [13]. We reasoned that in order to maximize fitness, males would try to attract females from greater distances and broadcast their size in relation to their competitive neighbors by maximizing the size of their pyramids and taking advantage of their larger body size to build higher and more prominent pyramids.

## 2. Material and Methods

Ghost crabs (Genera *Ocypode* and *Holpocypode* spp.) are considered to be reliable bioindicators of beach and environmental quality [22,23,24]. Ghost crabs are facultative scavengers, foraging on any form of organic material, but also predate other macro-invertebrates; and since they have no terrestrial competitors on the beaches and are able to endure long periods of starvation, are top predators in the simple food web of the sandy beaches [25]. Some of the display behaviors in the *Ocypode* species include air-borne sounds and substrate vibrations by either rapping or stridulating; visual courtship cues were male waving or chela-forward display; or petrified display-signals that males make from sand excavated from their burrows [18].

**Figure 1:**
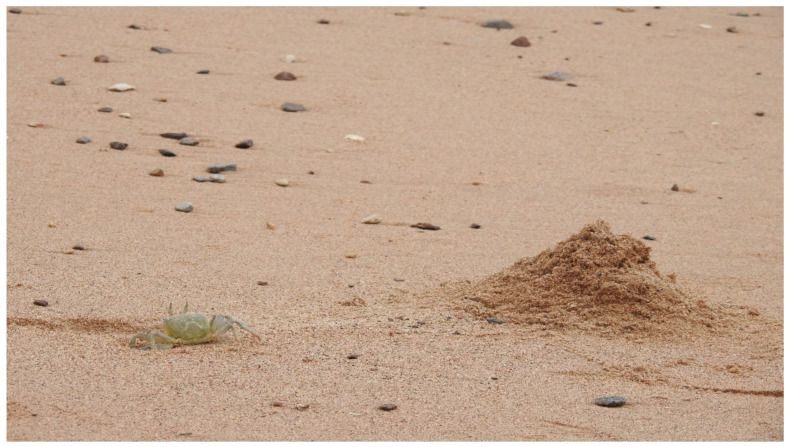
A typical Red Sea Ghost Crab (*Ocypode saratan*) burrow entrance with the sand pyramid on the outside. (Photo RY).

The RSGC is endemic to the Red Sea, and the only studies published to date on the species are from more than half a century ago [20,21]. Recently, RY and his high school students have initiated a study of the species [26]. They found that the RSGC is the most conspicuous member of the sandy beach communities in the Red Sea and that male burrow complexes consisted of a sand-pyramid (Figure 1), a pathway leading to it and around it, a vestibule at the entrance and a spiral burrow, which are only constructed by sexually mature adults. Physiological differences were noted in relation to body size by studying their branchial morphology, gill area and branchial volume in different-sized crabs [21]. RSGC was found to have fewer gills and less gill area than aquatic crabs, and the smallest crabs had the highest heart rates; blood characteristics indicated adaptation to semiterrestrial habitats and high ambient temperatures.

It was found that the pyramids are on average 16.5 (±2 cm, *n* = 100) high and the base circumference averaged 28 cm (±5, *n* = 100) wide [20]. For the construction of a new pyramid, a crab required an average of 1850 cm^3^ (±550, *n* = 45) of sand excavated from within the burrow and required about 80 min (±15, *n* = 25) to construct. He also suggested that the pyramids appear to be the sign-stimulus for attracting females, intruding males are fought off by the owner, and the nearest-neighbor-distance was 134 cm. In another study, it was reported that a male will guard his pyramid for 4–8 days, during which it does not feed [21]. Only when the female is in the vicinity of the pyramid do the males initiate vibration signals to convince the female to enter the burrow [21,27]. It was also reported that sexual selection by females is influenced by the fact that they choose to enter the burrows of like-handed males, i.e., the larger cheliped is on the same side in both individuals [20,26].

The study was conducted on the northeast beach in Eilat, Israel (29°32′34″ N, 34°58′39″ E to 29°32′33″ N, 34°58′41″ E). Eilat is situated in the northwestern corner of the eastern arm of the Red Sea. Owing to shore-development projects by the local municipality wherein gravel was used to layer the sandy shores for the sake of beach tourism, a short section of ca. 400 m still remains in its original, sandy substrate. This stretch is further divided into two sections—ca. 100 m of sandy beach for unregulated tourism and a further 300 m that extends eastwards into a military fortification and the international border with Aqaba, Jordan.

We conducted our study on the tourist section of the beach. At each session, we located all sand pyramids that were visible on the beach and measured their height (in cm), and the dimensions (height, breadth; in cm) of the burrow entrance. We assumed that the size of the entrance represented the relative size of the individual living in it, i.e., the carapace width [20,28,29,30,31]. Observations were made mostly in the morning hours, before the arrival of the tourists. Only those pyramids that were not disturbed by humans were measured. All observations were conducted during the months of March, April and May 2020. We conducted a total of 95 hrs of observation during which we successfully measured 54 pyramids.

We analyzed relationships between entrance volume (as relative size of the individual) and the heights of the pyramids as linear and/or quadratic tendency. In the first step, both variables were standardized, and the mean value was used as a reference point:

Z_ent/pyr_ = x − µ

where:

x—not standardized variable;

µ—mean value calculated from population.

Thus, the standardized variable means how much of the entrance volume/height of pyramids deviates from the average of the whole population. We then described the relationship of both variables using the equation of a linear and a quadratic function, where the standardized height of the pyramid was used as the dependent variable. Next, we performed Mandell’s test to check which model better described our relationship. We also calculated an extreme of quadratic functions according to the equation:

y = ax^2^ + bx + c; E = −b/2a

which indicates to what volume of the entrance the height of the pyramid increases and vice versa from what volume of the entrance the height of the pyramid decreased. All calculations were performed in R 4.0.3 [32].

## 3. Results

**Figure 2:**
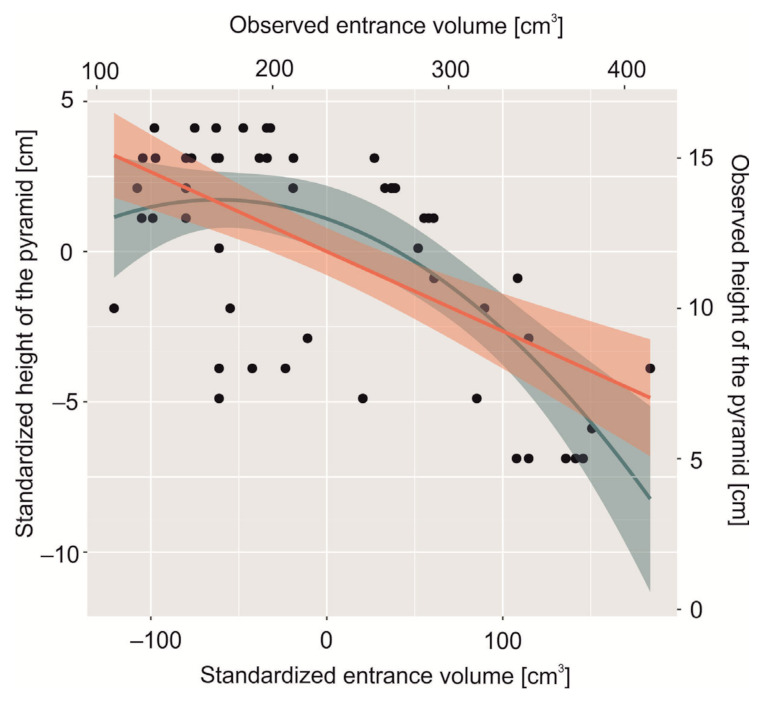
The linear (Red line) and quadratic (green line) model which shows how height of pyramid is related to the volume of the entrance to the burrow of the Red Sea Ghost Crab (*Ocypode saratan*).

The mean (± SE) entrance volume and height of the pyramid were respectively 230.8 ± 11.7 cm and 11.8 ± 0.49 cm, resp., *n* = 54. We found a negative significant correlation between these variables for both raw and standardized variables: r = −0.60, *p* < 0.001, *n* = 54; r = −0.60, *p* < 0.001, *n* = 54. The beta-coefficient (± SE) for linear relationships was −2.64 ± 4.82 and was different from 0 (t = −5.33, *p* < 0.001), and R^2^ for this relationship was 0.36. For quadratic function, the beta-coefficient (± SE) was −1.65 ± 6.06 and also was different from 0 (t = −2.72, *p* = 0.008), but in this case, R^2^ was higher than for linear regression and was 0.42. Mandel’s test to check which model is better suited to the data was significant (F = 7.39, *p* = 0.008), which suggested that the quadratic function better describes our relationship than the linear model. The extreme of the entrance volume was 169.01 mm, and after crossing this point of burrow entrance volume, the height of the pyramid decreased (Figure 2).

## 4. Discussion

The results of our study did not support our hypothesis. We had assumed that size would matter in that we expected to find a linear correlation between body size and pyramid height. We had predicted that the larger the male, the larger would be the pyramid. However, our results show the opposite in that the largest males in the population either built small pyramids or not at all; and the cut-off of the larger crab’s body size appears to be around 350 cm^3^. We discovered a step-wise function in the data in those crabs with the smallest body size of ca. 250 cm^3^ constructed the highest pyramids, with a declining tendency between 250–350 cm^3^, and extremely low pyramids beyond 350 cm^3^ (Figure 1).

We suspect the differences in the height of the pyramid between our research and those from more than half a century ago [20] result from the range of body size of RSGC in different geographical zones or that there is a seasonal or annual effect that has not been described to date. Further, the subject of anthropogenic disturbance could contribute to the subject of the height of the pyramids. We assume that frequent disturbance by beach tourism could influence the time and energy invested by a male ghost crab in the upkeep of his sand pyramid. Another point that needs to be addressed is the nearest-neighbor-distance and the parameters that influence burrow placement on the beach. In *O. ceratopthalmus,* on Seychelles, males only constructed copulation burrows around the full moon and only below the high tide mark [33]. This was not the case in our study, wherein all burrows and related pyramids are found throughout the moon’s cycle and also are above the high tide mark.

The results are in contrast to the SSS and the Handicap Principle, wherein it is always assumed that the larger, greater or more excessive the demonstration by the male, the better attraction of females and increased fitness. For example, female Southern Grey Shrikes (*Lanius meridionalis*) are attracted to males with larger larders, and in extreme conditions, even choose to be polygynous over monogamy, which is the norm in the species, as the mating system for the season [7,34].

In the past, the extreme dimorphism between the sexes and elaborate and diverse decorations of males even lead to mistakenly identifying them as separate species [35]. As previously mentioned, many of these extravagant displays were supposedly explained by the Handicap Principle [13] and supported by many a study [36]. The principle states that for signals/traits specialized for communication to be effective, they must be reliable, and in order to be reliable, they must be costly, and cost makes false signals unprofitable [37]. However, not only are there a wide range of different assumptions and theories that have evolved from the initial idea [38], there are also those who have found that the conclusions of the Handicap theory are incorrect and found no evidence for altruism or handicap [37,39,40]. Similarly, in our study, we find that the behavior of the male RSGC is actually contrary to what was expected if they were to conform to the principles of the Handicap theory. In our case, the largest were the smallest and with an inverse correlation with the smaller males building the larger pyramids. Although we have not evaluated the relative fitness of the different sizes of the RSGC, other studies of crustaceans substantiate the idea that female preferences are biased towards the larger individuals of the population [20,21]. Hence, our study does not substantiate the Handicap Principle and substantiates the findings of those who did not find evidence in its support.

The mating system in *Uca* spp. was classified as resource-defense polygyny in some species and as resource-free and promiscuous in others [19]. In our case, we assume that the proximity of the male pyramids to each other suggests a lek system and that the only resources the males seek are safe burrows for attracting females, i.e., resource-defense and promiscuous [15].

Early researchers concluded that in field conditions, the negative effects of burdening offspring with handicap genes outweighed the advantages of mating with a fitter male and, thus, disproved the Handicap Principle [41,42]. In our study, it appears that body size suffices to ensure fitness. We assume that the larger males are so prominent in the colony or are able to broadcast their size differently (auditory [27]) that they do not need to invest energy in building prominent pyramids or to patrol them against intruders [20,21] and care for their constant upkeep. One must also take into account that larger body size entails a greater investment in locomotion as compared to smaller congeners and may explain some of the discrepancies found in our study. Our study concurs with the study wherein the larger individuals derived higher benefits with lower investment as compared to smaller males [43]. It was also found that the sand structures are constructed only by males that are able to fend off intruders and that other conspecifics regarded the structures as indicators of the owner’s fighting potential [44].

## 5. Conclusions

In conclusion, we present the results of our study on display in Red Sea Ghost Crabs wherein the degree of investment in display-related sand pyramids is related to body size, but against the intuitive assumption that larger males build larger pyramids, we found that the smaller the male is the larger the pyramid it needs to build to try and obtain the attention of the vying females. However, our findings need to be further studied with a stress on the ambience and to elucidate whether the habitats differ in temperature, humidity, prey-base, etc., before concluding as to why the larger males desist from building pyramids. This study underwrites the importance of studying the mating systems of the macro-fauna of the beaches that are disappearing due to anthropogenic recreational activities and development.

## Data Availability

The data will be archived on Mendeley upon acceptance.
